# Measuring and increasing the brain health span across adulthood: a public health imperative

**DOI:** 10.1038/s41598-026-51403-3

**Published:** 2026-05-02

**Authors:** Lori G. Cook, Jeffrey S. Spence, Zhengsi Chang, Erin E. Venza, Aaron Tate, Ian H. Robertson, Mark D’Esposito, Geoffrey S. F. Ling, Jane G. Wigginton, Sandra Bond Chapman

**Affiliations:** 1 Center for BrainHealth ®, School of Behavioral and Brain Sciences, The University of Texas at Dallas, Dallas, TX USA; 2 Institute of Neuroscience, Trinity College Dublin, Dublin, Ireland; 3 Department of Molecular and Cell Biology, Helen Wills Neuroscience Institute, University of California, Berkeley, Berkeley, CA USA; 4 Department of Neurology and Neuroscience, School of Medicine, Johns Hopkins University, Baltimore, MD USA; 5 Center for BrainHealth, The University of Texas at Dallas, 2200 W. Mockingbird Lane, Dallas, TX 75235 USA

**Keywords:** Assessment, Brain health, Cognitive training, Executive function, Health promotion, Longitudinal study, Health care, Health humanities, Neuroscience, Psychology, Psychology

## Abstract

Extending *brain health* span – maintaining or improving cognitive, social, and emotional well-being – is critical to aligning health span with lifespan. This study examines 3-year outcomes from 3,966 adults (ages 19–94) in the BrainHealth Project, an online initiative integrating the BrainHealth Index (BHI) with cognitive training, lifestyle modules, and coaching. The BHI, assessed biannually, provides a multidimensional measure across factors of Clarity (cognitive function), Connectedness (social and purpose-driven engagement), and Emotional Balance (mental well-being). Results demonstrate sustained improvements in overall BHI and component factors, independent of baseline scores. Higher engagement with training tools – strategy-based learning, coaching, and brain-healthy habits – was associated with the greatest gains, underscoring the role of self-agency in brain health optimization. Improvements were observed across demographic groups, suggesting benefits regardless of age, gender, or education level. Findings support the potential for scalable, technology-driven interventions to help reduce years of cognitive decline while maximizing brain performance across the lifespan. Future efforts should focus on improving demographic diversity and retention as well as integrating precision brain health into public health initiatives.

**Trial registration:** ClinicalTrials.gov, NCT04869111 (registered April 27, 2021).

**Supplementary Information:**

The online version contains supplementary material available at 10.1038/s41598-026-51403-3.

## Introduction

In recent years, brain health has emerged as a major public health priority, driven by growing global efforts to prevent brain-based disease^1,2^. This attention is catalyzed by accumulating evidence of multiple modifiable risk factors for dementia, as highlighted in the 2024 Lancet Commission report^3^. While addressing risk factors is important, taking proactive steps to extend and strengthen brain health at every age may be even more impactful^4^. The need to promote healthy brain performance is underscored by evidence that neural and cognitive functions in healthy adults begin to decline as early as the late twenties – particularly brain blood flow and speed of mental processing – even in the absence of injury or disease^5,6^. In some scientific circles, it has even been postulated that brain aging itself might be considered a disease process^7–9^.

We propose that the more consequential question to address is whether holistic brain health can be measured and improved over time, aimed at countering whether the insidious loss of neural and cognitive health is inevitable. A recent report on research priorities from the National Academies of Sciences, Engineering, and Medicine^10^ noted that accelerating progress in understanding resilience to brain-based diseases such as dementia will require the dual approach of (1) quantifying brain health and (2) developing multi-pronged interventional approaches, ranging from individualized/precision medicine to scalable public health promotion strategies across the lifespan. Over the past one-and-a-half centuries, the human lifespan has doubled, and our health span has increased due to advancements in aspects such as heart health, physical and dietary health, the mitigation of infectious diseases, and improved living conditions^11^. There is now urgency to apply similar proactive efforts toward extending the *brain health* span – maintaining or improving overall brain health, starting young and continuing year by year – to ensure that continued gains in biological and chronological longevity are matched by prolonged cognitive, social, and emotional vitality.

Although various definitions of brain health exist, a growing consensus is emerging. *Brain health* encompasses a state of optimal neural development, cognitive function, well-being, and connectedness to people and to purpose^4,12^. Whereas ‘connectedness’ is a relatively recent addition to this definition, researchers and public health officials underscore its critical role in brain health^13,14^, positioning socialization (i.e., countering loneliness) and purpose as key contributors to brain health^15^. Thus, brain health is a dynamic interaction of cognition, social connectedness plus purpose, and emotional well-being, all of which work in tandem to contribute to thriving in daily life. Each of these three factors can be both a contributor to, as well as an outcome of, underlying neural health status. This bidirectionality is consistent with other multidimensional health concepts (e.g., physical health, where exercise both reflects and promotes health).

The potential to extend the brain health span, i.e., maintaining or increasing brain performance with age, is grounded in well-established research on neuroplasticity, demonstrating that the human brain retains immense capacity to adapt and change throughout life^16,17^. Although more research is needed to explore the extent of neuroplasticity that persists as humans age, there is consensus that the aging brain retains significant modifiability unless compromised by significant brain injury or disease. This runs counter to the widely held misconception that brain function is fixed at a young age and offers the opportunity to *improve* cognitive, social, and mental health functioning at any age through fostering practices to extend the brain health span^18^. This also runs counter to the belief that *overall* brain decline with aging is inevitable. Certainly, aging has been associated with declines in specific cognitive skills such as speed of processing and working memory, reflected in lower performance norms with increased age. However, even these skills can be enhanced with training^19^. Rather than accepting an unavoidable trajectory of loss, research is needed to determine the degree to which brain skills that are meaningful to everyday life functioning can be strengthened.

We propose that measuring brain health span requires a holistic framework that could reflect the interdependent contributions of cognitive, social, purpose-driven, mental health, and lifestyle factors. Because strengths or weaknesses in one domain can influence others, a multidimensional perspective offers a more integrative picture of how these factors together support daily functioning than comparing changes over time on isolated measures. Traditional screening tools and single-domain measures offer limited insights, as they fail to reflect these dynamic interactions across factors that shape brain health^20^. A wellness-oriented, integrative brain health metric is therefore essential – not only to monitor changes across domains, but to capture positive growth in neurofunctional capacity as well as early signs of decline over time^21^.

Recent advances include lifestyle-based composite measures, such as the Healthy Lifestyle Score^22^ and the Lifestyle for Brain Health (LIBRA) Score^23,24^, which assess modifiable risk factors such as diet, physical activity, and social engagement. While these measures are welcome advances, they remain largely deficit-oriented, primarily designed for at-risk or older populations, thus neglecting the importance of strengthening brain health earlier in life for the cumulative benefit of building reserve. They also typically offer only limited personalized guidance for fostering brain health practices across the lifespan.

In order to derive a holistic, wellness-driven, and dynamic/repeatable approach, our team developed and tested the BrainHealth Index (BHI), bringing together core measures of complex cognition with both novel and established assessments spanning daily life/lifestyle, mental health, social, and purpose domains to yield a multidimensional composite metric^25,26^. The BHI supports precision brain health applications by enabling both individuals and care providers to visually track personal brain health trajectories starting in early adulthood and continuing throughout life. Unlike traditional assessments that compare an individual’s performance against normative databases, the BHI functions as a personalized benchmark against which to measure improvement or loss rather than as a diagnostic classifier. Hence, the numerical index given to people is largely interpreted in comparison to *their own* progress and not in comparison to others. At baseline, individuals can explore the specific components that contribute to their overall performance, reflecting multiple pathways through which to support or strengthen brain health moving forward. The visualization also includes indicators as to the relative strength of each component for that individual. Based on these insights, individuals can then work with a coach to set meaningful goals addressing one or more of these brain health components (e.g., mental flexibility, sleep quality, engagement in social activities). This individualized approach parallels repeatable indices used in physical fitness, such as functional fitness batteries, which are often interpreted relative to an individual’s baseline and leveraged to guide personal goal-setting and track meaningful change over time in health promotion contexts^27^.

This proactive, optimization-based approach to brain health assessment represents a paradigm shift from a focus solely on deficits to one of growth, removing the fear and stigma of being told that one’s cognitive abilities are declining or are low compared to others. Instead, individuals are empowered to take ownership of their brain health, engaging with targeted strategies and practices to strengthen and sustain their brain health span, whatever their starting level. By charting an individual’s own degree of brain health modifiability/change, this approach provides a protocol to (1) measure the extent of individual neural and behavioral plasticity and (2) evaluate the degree to which age- or event-related bio-behavioral changes in overall brain health can be minimized or avoided – or determine for which cases these changes appear inevitable and unchangeable.

The seeming paradox in the statement that individuals may “have brain health without neural health” (M. D’Esposito, personal communication, January 28, 2025) is resolved by defining the health of the brain as a holistic measure combining clarity, connectedness, and emotional balance in the real world, which can exist and be improved despite underlying neural dysfunction. This approach permits a growth-perspective on brain health and applies even in the context of compromised neural health, such as observed in continuing recovery from concussion, stroke, chemotherapy-induced brain decline, depression, and other issues. Our previous research using randomized-controlled designs has shown evidence of improvements in brain health in all of these conditions^28–33^.

Just as the Framingham study informed a host of preventive approaches and health-promoting behaviors for cardiovascular function, large-scale longitudinal research is needed to establish proactive approaches for brain health, including establishing validated markers of brain health and identifying modifiable predictors^34,35^. In response to this need, the BrainHealth Project was launched in 2020 to address whether scalable, population-level assessments and interventions can harness neuroplasticity to enhance and sustain brain performance over time^12^. The BrainHealth Project aims to recruit 100,000 generally healthy adults over the first ten years, ranging from age 18 to late adulthood, to track brain health trajectories (i.e., brain health span) using the BrainHealth Index. Participants engage with coaching-enhanced online training modules designed to strengthen executive functions through adoption of cognitive strategies, further applied to social, emotional and lifestyle factors – including sleep, physical activity, and daily habits. The BrainHealth Project is a single-arm trial because: (a) its constituent strategy-based training interventions have previously been validated in several randomized trials, demonstrating causal efficacy, and (b) the virtual platform as a whole has been validated in recent studies^28–32,36–44^. The present study seeks to extend those findings into a much larger, more real-world cohort to inform population-level benchmarks for brain health and to examine the natural dose-response effects. Because the sample is meant to be large and inclusive, we can leverage natural variability in utilization to examine associations between degree of engagement and outcomes without random assignment. In essence, this longitudinal study reflects a hybrid effectiveness–implementation design^45^, evaluating the real-world impact of self-directed brain health intervention on participant outcomes while concurrently examining key implementation processes, such as utilization, adoption, delivery, and sustainability in naturalistic settings. The overarching goal is to determine how engagement with these tools can support and optimize brain function, whatever an individual’s context or starting level.

Importantly, randomized controlled trials (RCTs) require intensive interactions with trial evaluators and administrators that keep adherence rates much higher than is typically the case for “in the wild” interventions offered at scale to whole populations. A recent example is the US POINTER trial, which had a remarkable attrition rate of only around 11% but included multiple face-to-face meetings in their lifestyle interventions and required substantial staffing, infrastructure, and participant time commitment, all of which can dramatically increase the resource burden^46^. Translating RCT results into scalable, cost-effective programs can be even more complex. Even in standard clinical practice, pharmaceutical prescriptions for common physical conditions such as psoriasis and arthritis typically have low adherence rates of 30–35% after one year^47^. Hence in the BrainHealth Project, we expect even lower adherence, given a completely novel form of intervention for a new ‘condition’ – brain health. This is particularly true because the requirements for participation – extensive online assessment, coaching calls and engagement with online training – are more demanding of participant time and energy than simply taking a pharmaceutical. The artificial support of a randomized trial context would not have allowed us to assess the real-world viability and efficacy of an intervention with the capacity to be offered at very low cost to tens of millions of people.

Our group carried out an initial study to establish the feasibility of using a scalable online platform and repeatable, data-driven BrainHealth Index to measure individualized brain health trajectories as well as to deliver strategy-based executive function training, coaching, and healthy habit-building tools to promote gains^25^. Results from 180 healthy adults spanning ages 18–87 demonstrated the approach’s effectiveness, with 75% of participants exhibiting notable gain in their overall BHI score at 3-month follow-up, independent of age, gender, or education level. Importantly, the study highlighted a positive relationship between engagement in online training and BHI gains. This study provided promising evidence that adults of all ages can complete the online BHI, engage in proactive brain health strategies, and adopt brain-healthy habits in their life contexts through an online platform delivery, achieving measurable improvement in brain health and performance. A follow-up study of this cohort focused on mental health outcomes found that improvements in depression, anxiety, and stress symptomatology were maintained or continued to improve when assessed 6 months later, underscoring the need for expanded investigation of change trajectories over time^48^.

## Study Objectives

The current BrainHealth Project study reports on a large prospective sample of generally healthy adults followed over an interval of 3 years, drawn from the ongoing longitudinal study. The primary aim was to examine the impact of participation on holistic brain health, as measured by changes in the multidimensional BrainHealth Index (BHI), with biannual testing points every 6 months (from Time 1 – Time 7). Specifically, the major questions addressed by this longitudinal study were:

1. Could individuals across the adult lifespan improve brain health and maintain these gains over a 3-year period, given access to online brain health tools? How were changes reflected across time on the overall BHI score as well as on the three contributing brain health factors (Clarity, Connectedness, Emotional Balance)?
2. Who was able to demonstrate changes from the brain health training protocols as measured by the BHI, in terms of age, education, gender and starting level (BHI at baseline)?
3. What was the role of utilization – as demonstrated by completion of ongoing micro trainings, daily brain health habits, virtual coaching sessions, and/or accessing brain health resources – in achieving or failing to achieve gains on the BrainHealth Index?

### Methods

#### Study Design

The BrainHealth Project was designed as a prospective, longitudinal study, functioning as an interventional, open-label, single-arm clinical trial. This clinical trial was registered at ClinicalTrials.gov (Identifier: NCT04869111) on April 27, 2021. Following the initial pilot investigation phase^25^, recruitment for the longitudinal study began and will continue over at least 10 years of study, with no fixed “completion date,” similar to the Framingham Heart Study. Although trial registration occurred after initial participant enrollment commenced, all key eligibility criteria, outcomes, and analysis plans were defined a priori and are transparently reported. All study activities were approved by The University of Texas at Dallas Institutional Review Board (IRB) prior to participant enrollment, and all procedures comply with the Declaration of Helsinki. See the full published study protocol for an elaborated description of study procedures^12^. Overall BrainHealth Project recruitment and study procedures are still ongoing and being conducted in accordance with the standards provided by the IRB.

Primary procedures for the study are conducted online through a desktop computer or the BrainHealth^®^ app (the latter as of February 2024, available for both iPhone and Android users). At study enrollment and every 6 months thereafter, participants complete a BrainHealth Index (BHI) assessment. With each BHI completed, participants receive a high-level overview of their performance and can track their results over time. Participants can also schedule a personalized coaching session (every 3 months) to receive additional feedback and/or to address individual brain health goals.

Throughout the study duration, participants are encouraged to utilize the training in their online account; these consist of interactive micro-learnings related to optimizing brain health and overall well-being, beginning with a strategy-based cognitive training, SMART (Strategic Memory Advanced Reasoning Tactics)^33,80^ and including subsequent modules on sleep, stress, and practices that can support overall brain health in daily life. We collect and analyze participants’ engagement with their study account (e.g., frequency of logins, training completed, resources accessed, etc.) alongside the BHI outcomes. This allows us to determine how utilization of the online brain health tools may influence participants’ outcomes.

This 3-year report reflects a planned interim analysis, prespecified in the protocol and approved by the University of Texas at Dallas IRB, to provide benchmarks of feasibility, engagement, and early outcomes. The 3-year timepoint was selected because it provided sufficient sample size (nearly 4,000 participants with at least two assessment timepoints) and follow-up duration (up to 3 years) to meaningfully assess trajectories of change. Interim analyses are essential to: (1) continue to validate the BrainHealth Index in real-world, longitudinal samples, (2) refine predictive models, and (3) share early public health insights that can inform ongoing research and practice, similar to the way epidemiological cohorts (e.g., Framingham) release periodic analyses before full study maturity. Future reports will be determined based on data maturity and major follow-up and analytic milestones.

## Participants

The study targets generally healthy adults aged 18 years and older who are fluent in English, have regular access to an internet connection and device, and are able to hear and read information presented on a computer screen or mobile device. Recruitment occurs predominantly through word of mouth, online postings, social media, and advertisements. This includes outreach on the Center for BrainHealth website, its e‑newsletter, and research registries such as the Alzheimer’s Prevention Registry. The study team also posts on platforms like Facebook, Instagram, and LinkedIn. Study flyers and recruitment materials are additionally shared at in‑person and virtual events hosted by the Center for BrainHealth and by healthcare or community partners. Participants do not receive payment or reimbursement for taking part in this online study, and participants’ informed consent is obtained online through the BrainHealth Platform. Study exclusionary criteria are based on specific health conditions, including diagnosed neurodegenerative disease; a history of stroke, concussion, or brain injury that currently impairs their ability to function at their reported prior level (e.g., inability to carry out daily responsibilities); or a diagnosis of autism spectrum disorder without independent-level functioning. Other diagnoses, such as those representing other medical or psychiatric conditions and/or learning disorders, are captured through self-report in the context of ongoing study assessment timepoints and are not exclusionary, provided individuals are able to continue study participation without undue stress or frustration. Participants can discontinue their participation at any point during the longitudinal study period.

See Table 1 for a breakdown of participant demographics at baseline for this 3-year longitudinal sample. A total of 4,190 participants with at least two assessment timepoints were identified. Of those, 202 withdrew from the study, and 10 participants were excluded from the analysis due to invalid/no age entered. For gender, non-binary gender participants were not included in the analysis due to the low sample size (*n* = 12), which is not enough for statistically meaningful results. The final dataset comprised 3,966 participants, which were included in the statistical analyses.

Because enrollment was staggered and the study is ongoing, participant counts at each timepoint reflect multiple factors. At each assessment wave, participants were categorized as having either a *completed* (on time) assessment or an assessment which was *due but incomplete*. Importantly, participants remain eligible to complete subsequent timepoint assessments regardless of category, unless they have elected to withdraw from the study. Assessments that remain *due but incomplete* one year later are at that point classified as *drop-offs* and contribute to our estimates of attrition. However, given the study’s continuous, multi-year design, drop-offs may reflect episodic pauses in engagement rather than permanent attrition. See Table 2 for numbers of both completed assessments and drop-off counts at each timepoint across this 3-year study period. Lower drop-off counts at later timepoints also reflect the smaller proportion of participants who have reached those assessment timepoints, relative to earlier timepoints. For this study sample, attrition rates at individual testing timepoints were 20% or lower.

**Table 1:** Participant characteristics at baseline (*n* = 3,966).

Demographic variables				
Age Group	18–25 (*N* = 100)	26–45 (*N* = 413)	46–65 (*N* = 1639)	> 65 (*N* = 1814)
	n (%)	n (%)	n (%)	n (%)
Gender				
Female	66 (66.00)	278 (67.31)	1329 (81.09)	1351 (74.48)
Male	34 (34.00)	135 (32.69)	310 (18.91)	463 (25.52)
Race				
White	72 (72.00)	323 (78.21)	1473 (89.87)	1702 (93.83)
Asian	13 (13.00)	32 (7.75)	65 (3.97)	25 (1.38)
Black or African American	4 (4.00)	15 (3.63)	34 (2.07)	23 (1.27)
Native American/Alaska Native	0 (0)	1 (0.24)	0 (0)	2 (0.11)
Native Hawaiian/Pacific Islander	0 (0)	2 (0.48)	0 (0)	1 (0.05)
Other	6 (6.00)	18 (4.36)	25 (1.53)	27 (1.49)
Multiple races	5 (5.00)	22 (5.33)	42 (2.56)	34 (1.87)
Hispanic or Latino				
Yes	12 (12.00)	51 (12.35)	97 (5.92)	37 (2.04)
No	88 (88.00)	362 (87.65)	1542 (94.08)	1777 (97.96)
Education				
Pre Bachelor	32 (32.00)	52 (12.59)	202 (12.33)	246 (13.56)
Bachelor	44 (44.00)	163 (39.47)	666 (40.63)	563 (31.04)
Post Bachelor	24 (24.00)	198 (47.94)	771 (47.04)	1005 (55.40)
Annual household income				
Less than $20,000	22 (22.00)	16 (3.87)	43 (2.62)	53 (2.92)
$20,000 - $39,999	9 (9.00)	21 (5.09)	84 (5.13)	169 (9.32)
$40,000 - $59,999	8 (8.00)	35 (8.48)	126 (7.69)	240 (13.23)
$60,000 - $99,999	17 (17.00)	84 (20.34)	308 (18.79)	489 (26.96)
$100,000 - $250,000	31 (31.00)	185 (44.79)	701 (42.77)	580 (31.97)
More than $250k	8 (8.00)	63 (15.25)	297 (18.12)	128 (7.06)
Not Available/Prefer not to answer	5 (5.00)	9 (2.18)	80 (4.88)	155 (8.54)
Self-reported psychiatric diagnoses				
None	74 (74.00)	313 (75.78)	1355 (82.67)	1506 (83.02)
One	21 (21.00)	69 (16.71)	194 (11.84)	229 (12.62)
Multiple	5 (5.00)	31 (7.51)	90 (5.49)	79 (4.36)
Self-reported medical conditions				
None	88 (88.00)	374 (90.56)	1231 (75.10)	1323 (72.93)
One	9 (9.00)	28 (6.78)	294 (17.94)	358 (19.74)
Multiple	3 (3.00)	11 (2.66)	114 (6.96)	133 (7.33)

**Table 2:** Numbers of completed assessments and drop-offs during the 3-year study period across longitudinal timepoints.

Timepoint	Completed *N*	Drop-offs* *N*
T1	3966	-
T2	3966	-
T3	2501	806
T4	1663	210
T5	1153	53
T6	764	15
T7	478	2

*Note: Drop-offs are distinguished from those who are not yet due for their assessment at that timepoint and are defined as having an assessment remaining *due but incomplete* for longer than a year, based on the date assigned. Because of the study’s continuous, multi-year design, drop-offs may reflect episodic pauses in engagement rather than permanent attrition; data current as of sample date (March 1, 2025).

## Study Procedures

### Primary Outcome: BrainHealth Index (BHI)

The BrainHealth Index (BHI) provides a holistic, multidimensional assessment of brain health and performance. By integrating core measures of complex cognition with both novel and established assessments spanning daily life/lifestyle, mental health, social domains, and purpose, the BHI allows for both independent analysis of individual measures as well as a holistic view of their combined impact on overall function. Participants complete an online battery (average of 60–70 min to complete, in total) that includes cognitive performance tasks and self-report questionnaires.

The BHI yields four separate scores, shared with participants through their BrainHealth Platform dashboard, including: (1) an overall BHI score, along with scores for its three validated factors of (2) Clarity (cognitive health – readiness to reason through complex situations and create new solutions via executive functions, memory, and supporting functions such as sleep), (3) Connectedness (social health – perceived connection to people and purpose), and (4) Emotional Balance (emotional health – steadiness in adversity while remaining productive via reduced anxiety, enhanced mood, and decreased stress). These component scores are based on a factor analysis of change scores from measures shown in Table 3. Leveraging machine learning analytics rather than presupposed components, the composite score captures the interdependency of brain health dimensions. Furthermore, the three factors have been validated by neural metrics (namely, predictive hemodynamic response functions) obtained from a study cohort undergoing functional neuroimaging in conjunction with baseline, 6-month, and annual BHI assessment timepoints during their study course^42^.

The BHI was intentionally designed as a composite of both validated instruments and novel tasks developed and piloted in prior research at the Center for BrainHealth in both healthy and clinical adult populations. For the cognitive performance measures, the novel or adapted tasks included assessment of complex thinking abilities such as reasoning, abstraction, mental flexibility, and strategy, using multiple alternate-stimuli versions randomized across time points. Rather than evaluating single domains or basic competencies, such as attention, working memory, etc., the cognitive performance measures were designed to capture more integrated functions reflective of real-life demands and central to everyday information processing and communication^33^. The reasoning and abstraction metrics, in particular, have previously demonstrated robust sensitivity and specificity and have been proposed as tools to aid in identifying healthy aging adults as well as for monitoring cognitive health over time, providing early alerts to potential cognitive difficulties^49,81^. Questionnaires for the other domains of the BHI evaluate aspects of emotional well-being, quality of life, purpose, happiness, resilience, social support, fitness, and sleep, utilizing empirically validated tools where available. See Table 3 for a full list of measures included in the BHI.

**Table 3:** Measures included in the BrainHealth Index.

Measure	Assessment Instrument
Strategic Attention	Visual Selective Learning Task^82,83^
Abstraction	Proverb Interpretation Task (developed at the Center for BrainHealth, modeled after Uekermann et al^84^.)
Reasoning *Synthesis* *Interpretation* *Memory*	Strategic Cognitive Inventory (formerly Test of Strategic Learning - TOSL)^37,38,85^ Condensed synopsis of complex text (~ 550-word narrative) Fluency of take-home messages/interpretations from text Memory for text details (free and cued/elaborated recall)
Innovation	Fluency of high-level Interpretations from Picture Interpretation Task (developed at the Center for BrainHealth, modeled after semantic verbal fluency task, adapted from Lezak et al^64^.)
Processing Speed	Coding Task, modified from the Digit-Symbol Verification Task (DSVT)^86^
Sleep	Pittsburgh Sleep Quality Index (PSQI)^87^
Compassion	Questionnaire adapted from the Light Triad Scale^88,89^
Mood *Depression* *Anxiety* *Stress*	Depression Anxiety Stress Scale (DASS-21)^90^
Meaningful Activities/Purpose	Engagement in Meaningful Activities Survey (EMAS)^91^
Happiness	Oxford Happiness Questionnaire (OHQ)^92^
Social Support	Social Support Survey Index^93^
Resilience	Connor-Davidson Resilience Scale (CD-RISC-25)^94^
Life Satisfaction	Quality of Life Scale^95^
Social Engagement	Social BrainHealth Scale (developed at the Center for BrainHealth)^96^
Growth Mindset	BrainHealth Appraisal Questionnaire (developed at the Center for BrainHealth, based on Bandura^97^)
Fitness	Metabolic Equivalents: Cardiorespiratory Fitness (CFEQ)^98^

To enhance ease of use, assessments can be completed in shorter segments (with progress saved) over a two-week period at baseline and every 6 months thereafter. Individual scores are graphed to visualize progress over time, helping participants understand their brain health holistically (composite score) while identifying pathways for improvement (factor scores and component visualizations). Unlike traditional measures, the BHI evaluates changes (either gains or losses) against the individual’s own baseline, supporting a highly personalized approach.

### Brain health training: modules, habits, and resources

Upon completion of the baseline BHI assessment, participants access self-paced training modules, habits, and resources centered on the topics of brain health and holistic practices that can support overall brain health.

**Core Training**. Participants access micro-learning content, delivered in 5–10-minute daily-available units incorporating videos, knowledge checks, and personal application activities. The initial modules provide training in evidence-based cognitive strategies from the Strategic Memory Advanced Reasoning Tactics (SMART) protocol, with curricula adapted for digital delivery. Developed by researchers at the Center for BrainHealth of the University of Texas at Dallas, SMART focuses on improving executive functions through training of nine strategies supporting three core cognitive skill areas: (1) strategic attention (e.g., reducing information intake, prioritizing focus, and allowing brain downtime), (2) integrated reasoning (e.g., synthesizing information and applying abstracted ideas), and (3) innovation (e.g., considering multiple perspectives and possibilities, generating novel solutions). These strategies draw upon frontally-mediated brain networks and can be applied to everyday activities and responsibilities in support of cognitive, emotional, and social well-being^39,40^. SMART has also been shown through previous randomized trials to promote neural changes such as enhanced functional connectivity, cerebral blood flow, and neural efficiency^29,31,33^. Following SMART, training modules address stress management and resilience-building practices as well as sleep hygiene, integrating brain strategies with healthy lifestyle practices.

**Extended Training**. After completing the core training modules, participants access monthly themed micro-learning modules designed to reinforce the core training concepts and extend their application to broader areas of real-life application. Topics cover a broad range of applications, including aspects such as decision-making, gratitude practices, curiosity building, and fostering relationships, to name a few. Modules include interactive learning components, various media modalities, opportunities for personal reflection, and learnings about relevant brain-behavior relationships. Participants may also be introduced to complementary cognitive training approaches to further support skill development and application.

**Habits**. Each module is accompanied by a selection of specific and actionable habits to incorporate training concepts into daily routines. Just as modules range in focus from cognitive strategies to wellness or lifestyle practices, so do the habits. If a training segment focuses on single tasking (versus multi-tasking), a corresponding habit could be “Set aside 30- to 60-minute blocks of focused, uninterrupted time for your most important tasks today.” The BrainHealth Platform allows participants to receive daily habit reminders, track habit streaks, and earn digital badges.

**Resources**. A curated collection of publicly available educational content, including research, media articles, and video lectures, etc., that relate to holistic brain health is regularly updated to supplement participants’ existing knowledge base and support ongoing learning and engagement. While some individuals could have already engaged with similar content outside the study, the Resources section provides a structured, centralized, and thematically aligned collection designed to support the other intervention components, reinforcing the value of behavior change and cognitive strategies.

## Brain health coaching

Throughout the study, participants can engage in one-on-one or group coaching sessions. Participants can schedule quarterly (every 3 months) 20-minute videoconference sessions with study personnel who serve as brain health coaches. In these one-on-one sessions, coaches provide personalized feedback on BHI results, guide strategy application, and/or assist with goal setting. Individual session summaries are saved in participants’ dashboards for easy reference. In addition, study participants have access to monthly virtual group coaching sessions. These 45-minute group sessions use a workshop-style format, where coaches guide participants through personally relevant activities. These sessions aim to: (1) reinforce training concepts, (2) help participants develop specific next steps for applying the concepts in daily life, and (3) foster a shared sense of learning and community.

## Statistical analyses

For the primary outcome measure, the BrainHealth Index (BHI), we calculated means and standard errors for the general pattern of longitudinal change from baseline to 3-year assessments along with change statistics for each 6-month testing point interval. To assess whether longitudinal trajectories over three years depended on initial baseline level, four levels of baseline measurements were defined by quartiles. Average gradients beyond the baseline means were fitted by weighted regression, where weights were determined by the reciprocal of the variances of the means, and gradients were compared over the four baseline levels using *F*-statistics.

In addition to long-term change, we were interested in determining whether utilization (overall engagement with the platform) influenced the initial change from baseline and whether age, gender, or education level influenced potential differences among utilization groups. Utilization was defined by combining several categories - training modules completed, implementation of daily habits, participation in coaching calls, and number of resources utilized.

Since each of these categories was on a different numerical scale, we converted totals from each category to a rank score, based on a participant’s category score relative to the distribution of the full sample. That is, a rank score was calculated for each of the four categories of utilization from the quantiles of each distribution. More specifically, participants ranked 0 if the score was lower than 25% of the sample, 1 if between 25% and 50%, 2 if between 50% and 75%, and 3 if higher than 75% of the sample. Thus, the rank of each utilization category ranged from 0 to 3. Finally, the total utilization was computed by summing the four individual category ranks, ranging from 0 to 12; and these overall rank totals yielded the utilization group classifications Low (0–3), Modest (4–8) and High (9–12).

Index measures were dependent variables in a linear mixed effects model which included effects of time (T1/baseline and T2/6-months), utilization group (low, modest, high – defined above), age (continuous variable), gender (female, male), and all interactions involving time, group and age; all interactions involving time, group and gender; and all interactions involving time, group, and education. We modeled the error term as a variance component structure, a within-subject variance and a between-subject variance, since measurements within subjects are positively correlated. Our primary interest was the time/utilization interaction and whether age or gender influenced this interaction (i.e., both 3-way interactions).

A second tier of analyses, intended to assess the effect of utilization on index measures, considered only the sample of Low-level utilizers from the model described above. Over the course of the second six months (i.e., between T2/6-months and T3/12-months), some increased their engagement with the platform, switching to either modest or high utilization levels. From these new group designations (i.e., Low-> Low; Low-> Modest; and Low-> High), which were based on changes in utilization between T2 and T3, we ran the same mixed effects model as above and tested the time/utilization interaction, but for this analysis the outcomes were based on a T3-change from baseline (T3 – T1). All interaction contrasts from both models were assessed using *t*-statistics, and *p*-values were Bonferroni-adjusted.

## Results

### Longitudinal changes in brain health indices

**Fig. 1:**
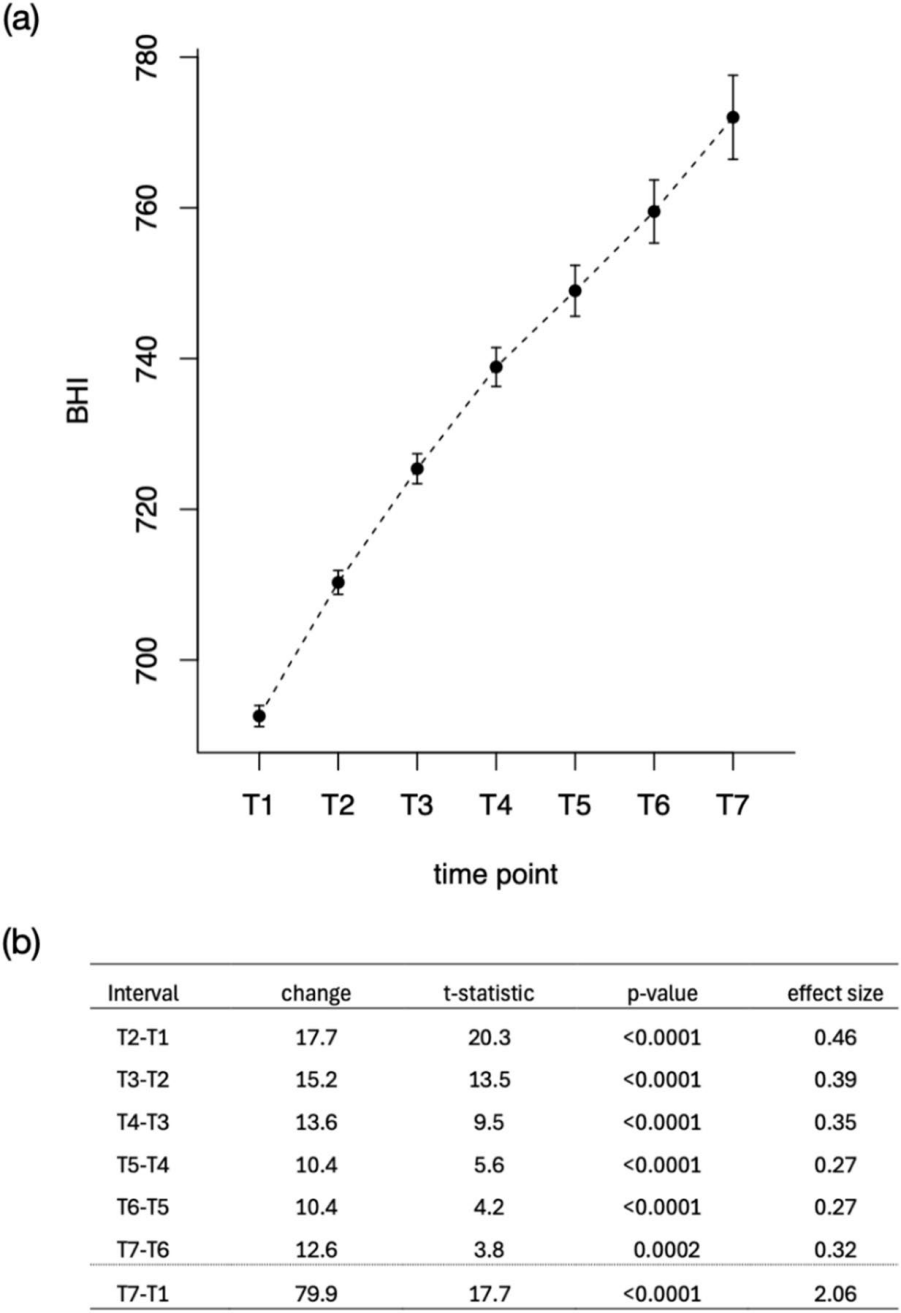
(a) Trajectory of the overall BrainHealth Index (BHI) score over the 3-year study period for the study sample as a whole, with (b) corresponding change statistics listed by testing point intervals - e.g., T2-T1 = Time 2 (0.5 years) – Time 1 (Baseline, 0 years). Effect size represents the change on a standard deviation scale (Cohen’s d).

**Fig. 2:**
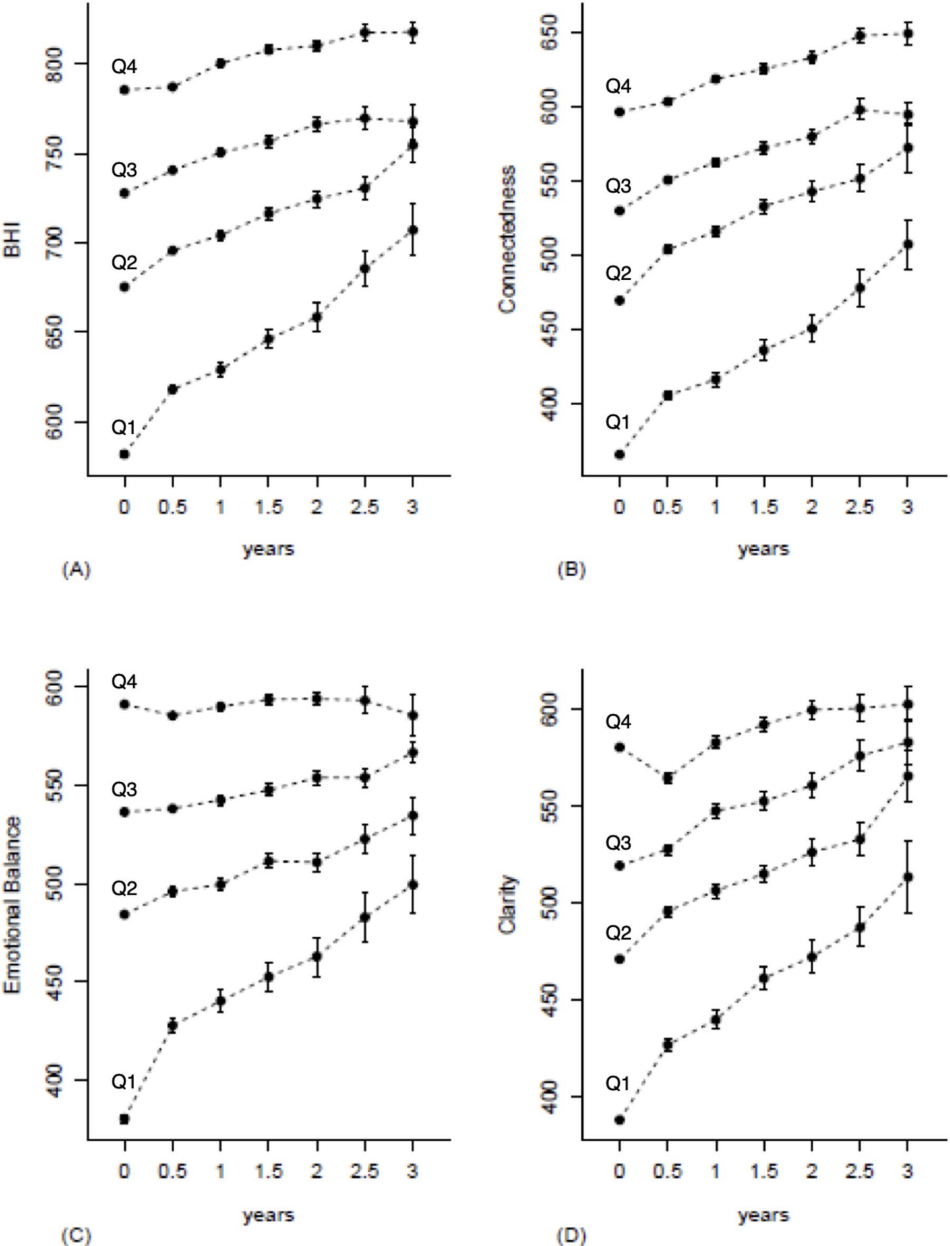
Trajectories of brain health indices over the 3-year study period, plotted by quartiles of initial baseline levels (Q1: 0–25th; Q2: 25th–50th; Q3: 50th–75th; Q4: 75th–100th percentile). Plots are presented separately for overall BHI (A), Connectedness (B), Emotional Balance (C), and Clarity (D) scores.

As seen in Fig. 1, the general pattern of longitudinal change for the overall BrainHealth Index (BHI) score is one of monotonically increasing change throughout the 3-year study period, with significant growth from one testing interval to the next for the study sample as a whole. When comparing baseline and 3-year timepoints (T7-T1), the overall gain was also highly significant, reflecting a very large effect size. Figure 2 shows that, with only a few exceptions, this general pattern of growth is seen regardless of initial baseline level. This was represented across the overall BHI score [Figure 2(A)] as well as for the three factors of Connectedness [Figure 2(B)], Emotional Balance [Figure 2(C)], and Clarity [Figure 2(D)]. See Supplementary Tables S1 and S2 for demographic and completed/drop-off assessment data reported across these baseline performance (quartile) groups.

For baseline levels in the lowest quartile, the longitudinal gradient is greater than that in the other baseline levels (BHI: *F* = 7.8, *p*=.002; Connectedness: *F* = 8.0, *p*=.002; Emotional Balance: *F* = 19.6, *p*<.001; Clarity: *F* = 2.9, *p*=.068) based on 3 degree-of-freedom *F*-statistics (see Supplementary Table S3 for gradient estimates). However, to make sure that these gradients could not be solely explained by regression to the mean, we removed the lowest quartile gradient and compared the gradients of the upper three baseline quartiles with 2 degree-of-freedom *F* statistics. With the exception of Emotional Balance, we see that they all have comparable gradients (BHI: *F* = 2.0, *p*=.163; Connectedness: *F* = 2.8, *p*=.092; Emotional Balance: *F* = 9.7, *p*=.002; Clarity: *F*=0.53, *p*=.597).

### Impact of utilization

**Fig. 3:**
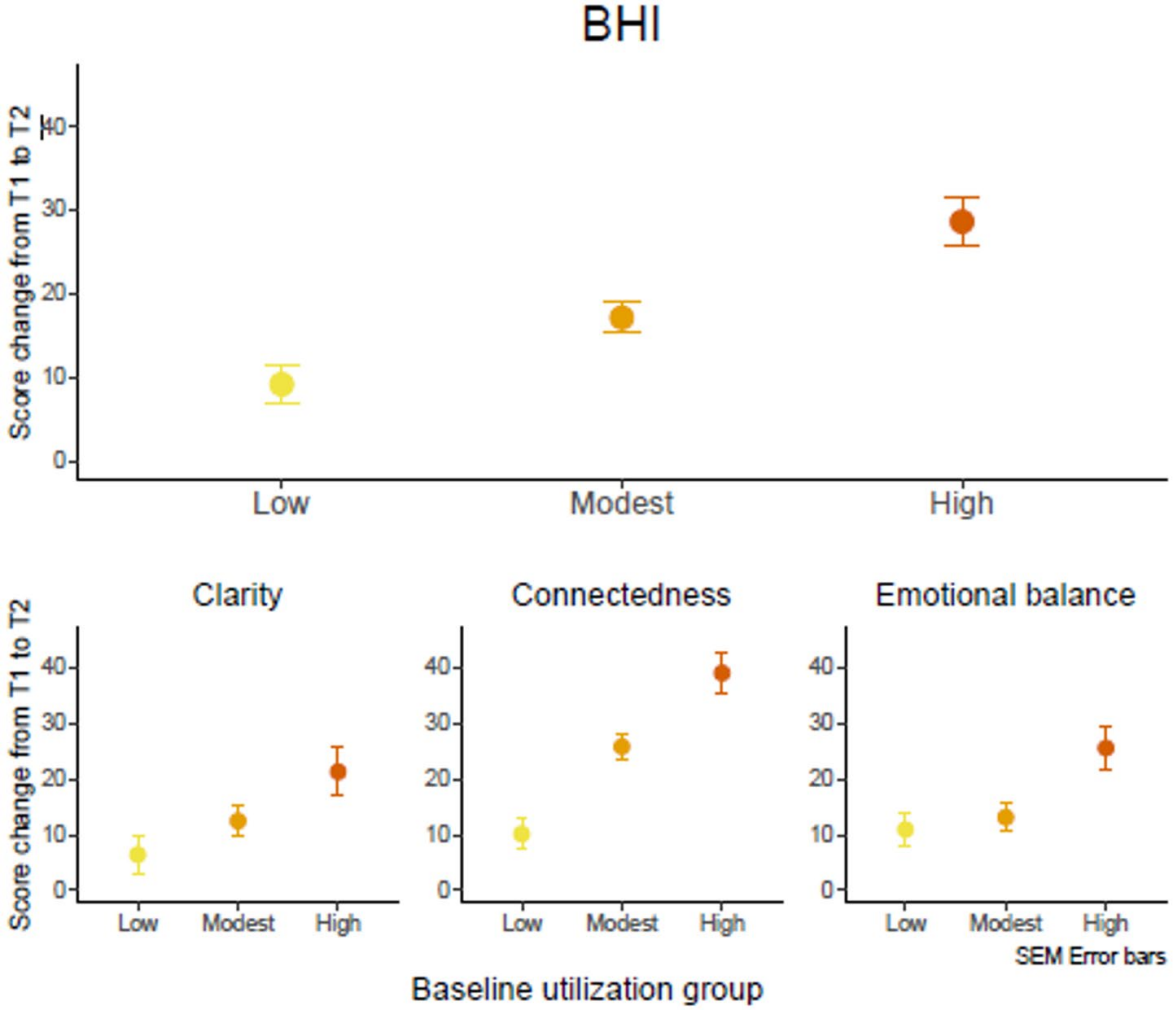
Magnitude of score changes from Time 1 (T1) to Time 2 (T2) assessments across all brain health indices, by utilization groups (low, modest, high).

See Table 4 for results from the linear mixed effects models addressing the effects of utilization on the change in brain health indices. Utilization had an impact on degree of change across all index measures. Figure 3 shows graded change across the three levels of utilization, with minimal change in those with low level of utilization and greatest change in those with high level of utilization. This interaction is significant for all indices (see Table 4). Notably, this general pattern of graded change as a function of utilization did not depend on gender or on age, as there were no significant three-way interaction effects between time, group, and gender or between time, group, and age on any of the scores. This suggests that changes in these scores across the utilization groups were not influenced by gender or age. Education did have a small effect, in that the modest utilization group demonstrated small increases in overall BHI score with increasing levels of education. Attrition rates and platform utilization were similar across the baseline performance (quartile) groups (see Supplementary Table S2 and Supplementary Figure S4, respectively).

**Table 4:** Tests addressing the effects of utilization on the change in brain health indices using linear mixed models.

			Overall BHI		Clarity		Emotional Balance		Connectedness	
Effects	ndf	ddf	F-statistic	*p*-value	F-statistic	*p*-value	F-statistic	*p*-value	F-statistic	*p*-value
Time: Group	2	3951	13.93	< 0.001***	3.43	0.033*	4.89	0.008**	20.27	< 0.001***
Time: Group: Gender	2	3951	2.09	0.12	0.27	0.76	1.41	0.24	2.68	0.07
Time: Group: Age	1	3951	0.13	0.88	0.04	0.96	0.10	0.91	1.20	0.30
Time: Group: Education	4	3951	2.29	0.06	1.07	0.37	1.64	0.16	1.75	0.14
Time: Group change (T1 to T3)	2	477	6.52	0.002**	5.78	0.003**	0.97	0.38	6.00	0.003**

Notes: Group = utilization category; * *p* <.05, ** *p* <.01, *** *p* <.001.

Post-hoc analysis using pairwise comparison (see Table 5) showed that, compared to low utilization, high utilization led to a significantly greater change between Time 1 and Time 2 (6 months) in the overall BHI score, as well as in the scores for Clarity, Emotional balance, and Connectedness. Modest utilization resulted in a significantly greater change in the overall BHI score and the Connectedness score, compared to low utilization. High utilization also led to significantly greater BHI score change than modest utilization. These results suggested that score changes from T1 to T2 varied depending on the level of utilization. The mean score changes across the board are lowest for low utilization and highest for high utilization. The overall BHI and Connectedness scores exhibited more dramatic score differences among the groups than those for Clarity and Emotional balance (see Table 5).

**Fig. 4:**
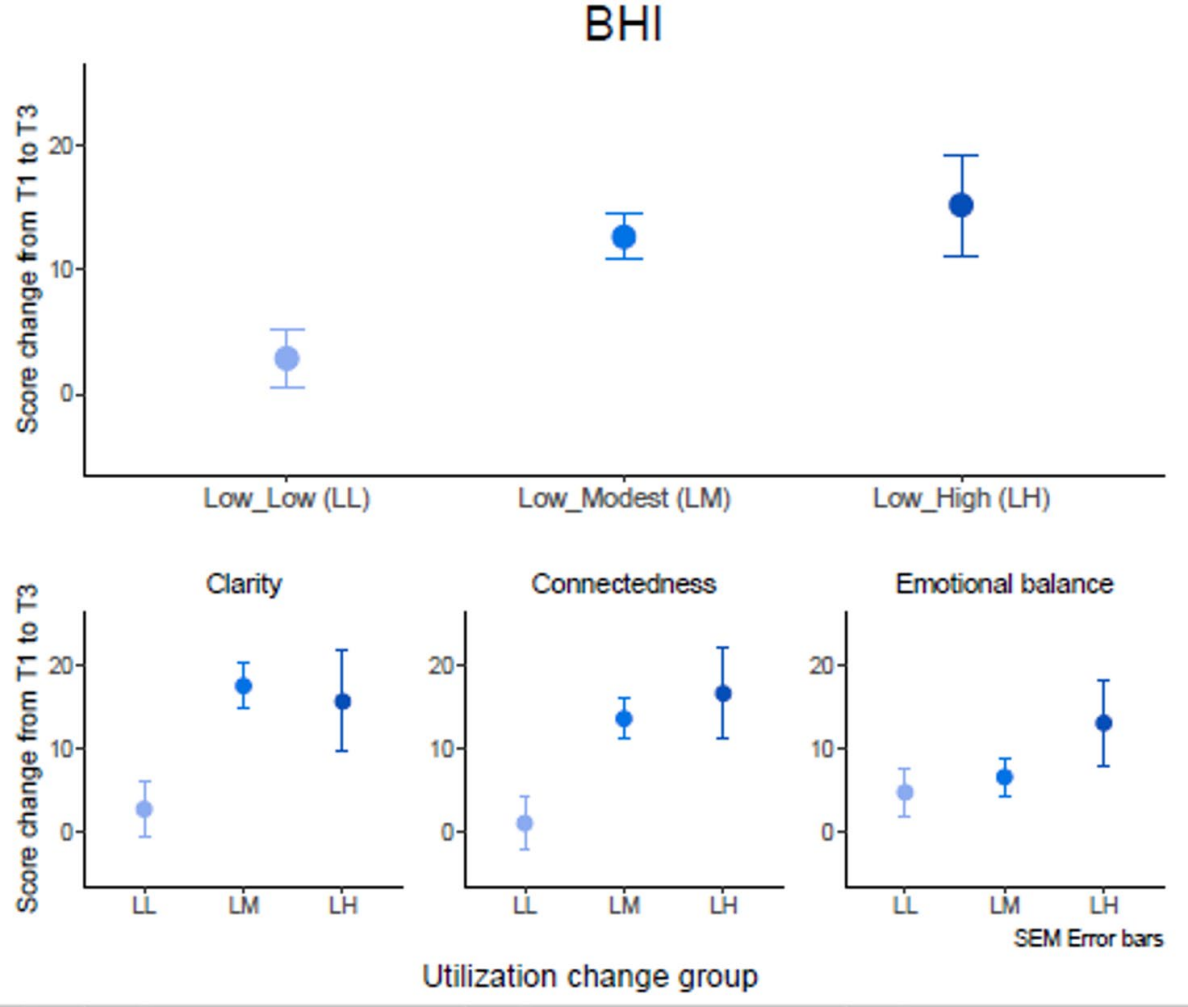
Magnitude of score changes from Time 1 (T1) to Time 3 (T3) assessments across all brain health indices, considering only initially low-level utilizers.

Tables 4 and 5 also show the effect of utilization change over time for those who were initially low-level utilizers. Those who switched to higher-level utilization over the following six months had higher mean changes on the indices through one year (see also Fig. 4). Conversely, those who remained low-level utilizers had no change on their brain health indices through one year.

**Table 5:** Results from post-hoc analysis using pairwise comparison, including utilization group contrasts of T2-T1 change (6 months) and low utilizers’ T3-T1 change (1 year).

Utilization group Contrasts of T2 – T1 Change	Overall BHI			Clarity			Emotional Balance			Connectedness		
	t-statistic	Bonferroni-adjusted *p*-value	effect size	t-statistic	Bonferroni-adjusted *p*-value	effect size	t-statistic	Bonferroni-adjusted *p*-value	effect size	t-statistic	Bonferroni-adjusted *p*-value	effect size
High – Low	5.36	< 0.001***	0.46	2.76	0.017*	0.24	3.02	0.008**	0.26	6.19	< 0.001***	0.53
Modest - Low	2.79	0.016*	0.19	1.43	0.39	0.10	0.57	0.92	0.04	4.28	< 0.001***	0.29
High - Modest	3.40	0.002**	0.27	1.76	0.22	0.14	2.75	0.018*	0.22	3.04	0.007**	0.24
Utilization Group change Contrasts of T3 – T1 Change	*t-statistic*	*Bonferroni-adjusted p-value*	effect size	*t-statistic*	*Bonferroni-adjusted p-value*	effect size	*t- statistic*	*Bonferroni-adjusted p-value*	effect size	*t- statistic*	*Bonferroni-adjusted p-value*	effect size
Low_High – Low_Low	2.64	0.025*	0.29	1.86	0.018*	0.18	1.39	0.42	0.15	2.51	0.037*	0.28
Low Modest – Low_Low	3.30	0.003**	0.23	3.35	0.003**	0.24	0.48	0.95	0.03	3.18	0.005**	0.22
Low_High – Low_Modest	0.58	0.92	0.06	−0.28	0.99	0.03	1.14	0.59	0.12	0.51	0.94	0.05

Note: All *p*-values are Bonferroni-adjusted; * *p* <.05, ** *p* <.01, *** *p* <.001.

### Four case illustrations

**Fig. 5:**
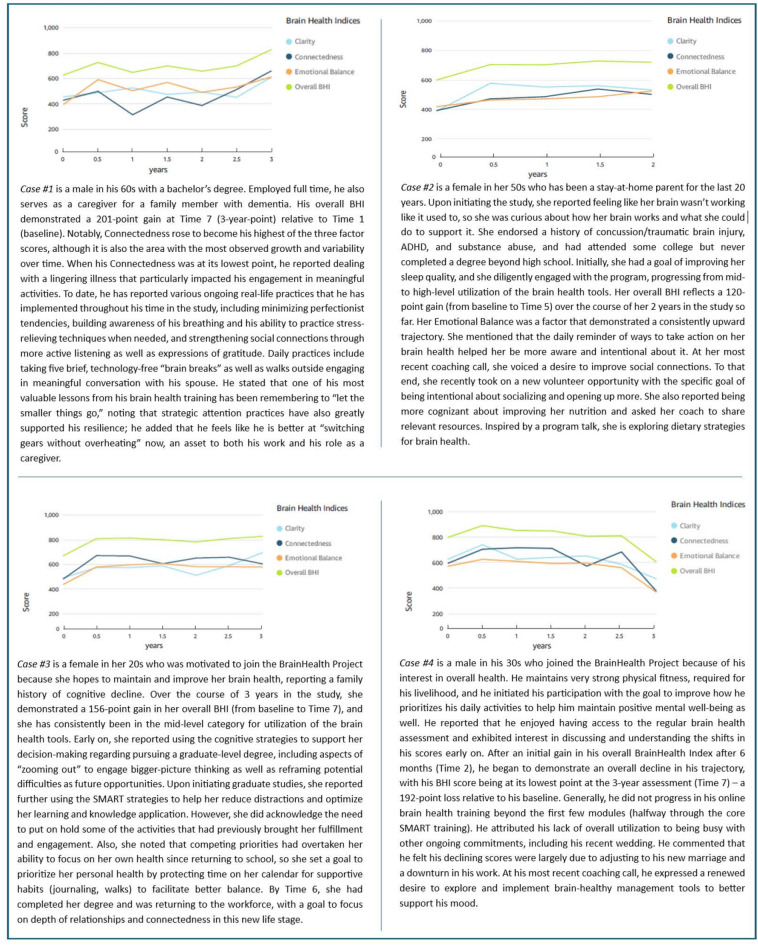
Case illustrations, including patterns of: dynamic changes reflecting upward growth (Case #1), general longer-term maintenance (Case #2), episodic declines in particular contributing subcomponents (Case #3), and a downward course of brain performance (Case #4).

Figure 5 presents four brief case examples which highlight the dynamic nature of individual brain health trajectories and demonstrate how the brain health factors can guide individuals in setting goals to maintain an overall BHI score. By linking these insights to personalized goals, the cases illustrate the utility of the BrainHealth Index in promoting specific, actionable strategies for sustained growth and optimization across varying life contexts. These cases underscore the distinctive insights derived from the BrainHealth Index, enabling a precision approach to brain health.

## Discussion

This study evaluates the scalability and efficacy of the BrainHealth Project, a longitudinal initiative designed to measure and enhance brain health through a multidimensional online platform, i.e., the BrainHealth Platform^12^. Over a three-year period, data were collected from a diverse cohort of adults (ages 19–94) across all 50 US states and over 60 countries with biannual (every 6 months) assessment using the BrainHealth Index. The platform also provided secure, remote access to brief, evidence-based micro-trainings targeting cognitive clarity, social connectedness, and emotional balance.

Findings demonstrate significant improvements in holistic brain health, with gains observed across cognitive, social, and emotional domains. These results extend prior work by Baker et al.^45^, which showed cognitive benefits in older adults following structured lifestyle interventions. Unlike their in-person model, the BrainHealth Project offers a scalable, cost-effective alternative that reaches individuals across the adult lifespan, including younger adults not yet at risk for dementia.

Importantly, this study challenges the prevailing narrative of inevitable cognitive decline, suggesting instead that brain health can be proactively cultivated at any age. This perspective is reinforced by Attarha et al.^19^, who reported improvements in anterior cingulate acetylcholine levels in older adults following online cognitive training. Together, these findings advocate for a paradigm shift toward lifelong brain health optimization through accessible, multidomain interventions. The implications of our current study findings for extending healthy brain function are summarized below, informing (1) *what* is possible in terms of measuring and enhancing brain health, (2) *who* is likely to benefit from this proactive brain health approach, (3) *how* improvements were likely achieved, and (4) *why* brain health promotion is a promising pathway to support overall health outcomes.

### *What* can measure and promote increase in brain health span

First, this study demonstrated that the online BrainHealth Index is sensitive to measuring and tracking changes (gains and losses) in group and individual brain health trajectories over time. In a large prospective sample of adults ages 19–94, the majority of participants who took advantage of accessing and engaging with the brain health tools and strategies showed significant gains over three years. Nonetheless, we acknowledge that some individuals showed losses (See Case #4) or maintained performance over time after initial gain (see Case #2). Our ongoing and prior research suggest that the BHI can be sensitive to the earliest signs of decline – detecting reduced baseline scores and slower training gains among individuals with subjective cognitive complaints (unpublished data) and successfully distinguishing healthy controls from those with mild cognitive impairment or early Alzheimer’s disease using BHI sub-measures^49^. With the addition of richer longitudinal data and machine learning analytics, we aim to create a precision triage system that supports early identification and timely intervention.

In terms of gains, the current data suggest the BHI was informative in evaluating the efficacy of ongoing *online* delivery of micro-learning brain health modules, with comparable results to those previously reported^25^ as well as those achieved through previous studies utilizing *in-person* training delivery^28,29,32,33,36–40,43^. Additionally, the BHI showed viability in capturing the **complex**,** multidimensional nature of brain health**^20^, with upward trajectories observed in both the holistic score as well as its three contributing factor scores (Clarity, Connectedness, and Emotional Balance). These trends were visualized at the group level (Figs. 1 and 2) as well as illustrated more dynamically at an individual level (Fig. 5), supporting its use in precision brain health.

Both the composite BHI score and its factors provided valuable insights regarding the course of change, as demonstrated by the case examples. These measures showed sensitivity to charting dynamic changes reflecting upward growth (Case #1), general longer-term maintenance (Case #2), episodic declines in particular contributing subcomponents (Case #3), and a downward course of brain performance (Case #4). By allowing users to visualize the tracking of their personal brain health performance scores through an accessible platform, the BHI can serve as a motivational tool to nudge habit change with simple steps to meet personal goals. For example, some users may aim to improve brain health, whereas others may be happy to maintain their current brain performance, while others may seek to counteract losses in a timely manner.

The BrainHealth Index’s utility extends beyond individual tracking for the impact of the SMART training offered by the BrainHealth Project; it offers a promising metric for evaluating other brain health interventions as well. Recent investigations reveal the BHI’s sensitivity to cognitive training using BrainHQ^31,44^ and Menopausal Hormone Therapy^50^. Other efforts are underway to evaluate the sensitivity of the multidimensional Index with its subcomponents to measuring gains from approaches such as neuromodulation or lifestyle interventions such as physical fitness training. Future efforts should continue exploring its potential applications in medical, mental health, and preventive care contexts.

One paramount question to address is what aspects contributed to the observed brain health gains. The present findings demonstrate that sustained improvements were achievable, regardless of an individual’s starting level, when given continual and easy access to brain health training, content, and practices. Participants in each quartile, based on baseline BHI, showed incremental gains over time, without evidence of ceiling effects. We propose that the strategy-based executive function training (SMART) equips participants with metacognitive tools that could be applied to support meaningful, real-life demands to facilitate ongoing improvements. SMART has shown to promote generalized gains by engaging higher cognitive functions applicable across various contexts^28–31,33,40,51^. Evidence from SMART and similar programs demonstrate broad benefits, including enhanced executive function, psychological well-being, and neural connectivity, particularly within the brain’s frontal networks, across both clinical and non-clinical populations^28–31,33,36–42,51^. Strategy-based interventions stand out for their ability to generalize to multiple domains of daily function^52,53^, whereas bottom-up approaches, such as speed-of-processing training, tend to show domain-specific gains^54^. These findings highlight the potential for integrating top-down and bottom-up approaches to tailor brain health interventions to individual needs and optimize outcomes across various life stages.

### *Who* benefits from the proactive brain health protocols

Notably, overall brain health gains in this study cohort were independent of age, education level, gender, or starting level of brain health performance, yielding two somewhat unexpected findings. First, we found that younger people generally benefited as much as older participants. This challenged a commonly held perspective that older individuals would likely benefit more, given that they have typically experienced more decline, and young adults would have little to gain. This similarity reinforces the idea that adults can optimize their brain health at any age and supports the recommendation to begin proactive brain health practices early in adulthood and continue throughout the adult lifespan. As with physical health, it is easier to preserve and build brain function starting at younger ages than to regain lost ground after substantial decline has occurred.

Second, we found that those in the lowest BHI performance quartile at baseline made greater gains over time than those in the upper quartiles (Fig. 2). While it is likely that this finding could possibly represent some statistical regression to the mean, it is clear that lower baseline brain health can be improved to narrow the gap with those with higher baseline levels. Many would say that this is intuitive – given that generally those with the greatest room for improvement often show the biggest changes. Nonetheless, there is a common misconception that lower performance from the onset is somehow fixed in nature and thus linked to poorer long-term outcomes and limited ability to close the gap to those with higher performance. The results of greater gains in lower performers over the three years speaks to the likely contribution of the brain’s inherent neuroplasticity and its capacity to continuously improve and be strengthened, given proper practices^17,55^. Moreover, for those with higher baseline performance, their changes over time did not decrease linearly with baseline BHI scores, as would be predicted by regression to the mean. In fact, those in the highest quartile showed statistically comparable gains to the second and third quartiles. The only exception to this is for the Emotional Balance factor, which may require more intensive interventions to achieve gains equivalent to those seen for the Clarity and Connectedness factors.

We were not surprised by the evidence that females and males showed comparable gains based on utilization. Nor was the finding of no significant educational effect on gains based on utilization unforeseen, given that we had a relatively well-educated group, with less than 15% having lower than a bachelor’s degree-level education. These gender and education findings are also consistent with prior findings from our first study cohort^25^. The relatively high education of our group highlights one major limitation of the present study, in that the results may not generalize to less educated individuals. We are actively working to recruit more participants from less educated backgrounds.

In sum, our results lend support to the universal applicability of proactive strategy-based executive function training to promote brain health, at least in those with a high school education or higher. The comparable gains across age groups and education levels represented in our sample suggest that key demographic factors which often predict disparities in health outcomes and/or responsiveness to health-focused interventions may not face the same barriers when it comes to increasing brain health span^56^. From a public health perspective, these findings reinforce the potential for brain health strategies to benefit most populations, regardless of starting level. Still more research is needed to replicate these findings in more diverse populations.

### *How* can brain health gains be achieved

In this single-arm design study, we believe that practice effects (i.e., repeated exposure to the BrainHealth Index) as an explanation for the observed improvements can be ruled out for the following two reasons. First, participants with generally low utilization who repeatedly completed the Index did *not* show significant gains. Second, we have shown in previous research that taking the BHI multiple times without engaging in the training did not yield similar improvements to those in the current study^25,44^.

Another potential confounder that can likely be ruled out is that participants improved simply due to non-specific factors, such as halo effects of study participation, the mere attention given to them, or expectations that this trial would improve their brain health, regardless of specific training. These explanations are countered by evidence from prior clinical trials of our cognitive training where participants in active control conditions (e.g., psychoeducation or physical exercise training) did not improve on measures from the major Clarity factor of the BHI when given equal attention^29,36,37^. These findings reinforce the conclusion that active skill- and habit-building, rather than passive expectations, drive meaningful brain health gains. Moreover, while they may contribute to early gains, the sustained improvements observed over multiple years suggest that effects are not solely attributable to novelty or attention.

One potential mechanism of gain is that of a direct effect of specific metacognitive strategies and associated habits in allowing participants to meet their daily life demands, combined with implementing advice about nurturing social connectedness and maintaining emotional balance to drive cognitive resilience. The four case studies (Fig. 5) give distinct individual examples of such a mechanism. More generally, higher utilization entailed consistent engagement with the online tools – assessments, coaching sessions, strategy-based micro-learnings, and habit-tracking – and was associated with greater gains in brain health over the 3-year follow-up period. These tools were designed for “bite-size” delivery, requiring minimal time (no more than 15 min within a single day), to motivate continued use and to optimize overall learning and application.

Encouragingly, utilization was not static: among participants with low utilization in the first 6-month cycle (*n* = 507), 63.12% went on to increase their engagement over time (i.e., shifted to a higher utilization category). This increase in utilization further supports the strategy-and-habit-learning hypothesis rather than a more non-specific global expectation mechanism to explain the improvements in brain health. We cannot rule out that changes in both utilization and the Brain Health Index may each have arisen because of a third factor – increased motivation or curiosity/personal interest – rather than being causally connected, as these can be potential confounders in any self-directed program. We embrace this as an important factor. In fact, the platform, including coaching and habit reminders/nudges, is specifically designed to motivate as well as to instruct. Hence the mere availability of an attractive and usable platform offering the expectation and pathway to improved brain health may drive this very motivation we invoke as a hypothetical third factor. Future efforts should elicit the degree of motivation and curiosity at different time intervals to see how these influence utilization and performance. Importantly, our large sample and longitudinal design will enable future modeling of these individual differences, helping to disentangle intrinsic motivation from intervention effects.

One of the most plausible explanations concerns the possibility that learning strategies to control one’s brain function and brain health makes participants feel more *empowered* and *in control* – in other words, they develop greater *self-agency.* This explanation is entirely compatible with the previous one regarding the metacognitive strategies themselves. Indeed, we consider both the utilization and implementation of specific strategies and habits, leading in turn to increased feelings of self-agency – and continuing in a mutually reinforcing cycle – as being the most likely mechanisms underlying the observed 3-year improvements in brain health. Research shows that simply inducing a person to think abstractly increases their sense of power^57^. Central to our training are metacognitive techniques such as “zooming out” from a particular situation or problem to widen one’s perspective and take a more abstract view of it. This is a technique used, for example, by Case 3 (Fig. 5). Metacognitive strategies are the essence of abstract thinking, and we hypothesize that engaging in these strategies increases empowerment and hence a sense of self-agency.

We interpret the observed strong relationships between utilization and improvements in brain health as likely being due to the mutual reinforcement of utilization of key strategies and habits, leading to an increasing self-agency. As such, increases in self-agency works to further boost utilization, as shown by the low utilizers shifting to higher levels of utilization. By this argument, utilization is a form of self-agency in action. The executive function skills learned during the SMART training – which have been shown to improve brain health in previous, smaller randomized controlled trials^28,29,31,33,36–40^– give participants self-agency over their own attention, thought, and emotion processes. This sense of control over their own brain processes should increase self-agency more generally. Self-agency kindles a sense of choice, which increases motivation via activation of dopamine-linked reward networks in the brain^58^.

In support of this explanation, research shows that cognitive training increases self-agency in older people. A recent study of over 12,000 older U.S. adults found that individuals with greater sense of control at the onset of the study had improved physical health outcomes over the study’s 4-year follow-up period^59^. Moreover, a stronger sense of control was associated with greater engagement in health-promoting behaviors as well as higher outcomes in multiple aspects of well-being and social connectedness^59^.

However, simply knowing which actions to take – through awareness and education, for example – is insufficient to drive the lasting behavior change necessary to reduce risks and promote health^60,61^. We propose that a key component of self-agency is its link to utilization of potent metacognitive strategies for controlling brain processes – what we have called *self-agency in action*^62,63^. This ability to translate intention into action is intrinsically linked to executive function, a set of cognitive processes that enables individuals to regulate thoughts, actions, and emotions in pursuit of goals^64,65^, and which is trained in the current project’s online SMART program. Importantly, executive functions can be strengthened through training and, as our data suggest, can result in improved brain health over an extended period of time^65,66^.

These findings motivate further research and clinical efforts to explore strategies for fostering self-agency in brain health promotion. We propose that individuals are more likely to show sustained improvement when they (1) perceive that they can impact their brain health, (2) are empowered to take action, (3) are equipped with executive function tools and (4) have ready access to technology-driven nudges to continually reinforce and practice brain health strategies and habits. Importantly, low utilization of the brain health tools in our current study could not be explained by lower baseline performance. Indeed, those in the lowest baseline quartile manifested the highest gradient in enhancing their brain health span. Further research must unpack the triadic relationship between executive function, strategy-and-habit utilization, and self-agency so that the potency of brain health training can be further increased. Moreover, because the BHI includes individual measures related to growth mindset and life satisfaction, future investigation could examine their influence on these relationships (e.g., do individuals with higher growth mindset scores at baseline demonstrate greater initial training utilization and/or do participants who experience BHI gains show corresponding increases in growth mindset over time?). Just as has been shown in physical health, individual self-agency – in symbiotic relationship with executive function and utilization – is likely a pivotal factor in maintaining and improving brain health, fostering autonomy and resilience in navigating daily challenges.

### *Why* brain health promotion is imperative

The primary health significance of this study is its demonstration that the complex and multidimensional construct of “brain health” can both be longitudinally measured and enhanced, along with its contributing factors, across the adult lifespan, in independently functioning individuals who are relatively healthy or may have some degree of compromised brain health. The current findings motivate efforts to integrate brain health metrics into clinical and research settings to advance health promotion strategies with metrics relevant to everyday life. In sum, the BHI shows promise as a novel, validated, change-sensitive tool capable of tracking both growth and early detection of decline in brain health over time, filling a void in available brain health measures. Not only is the BrainHealth Index holistic with its contributing subfactors, but it is also scalable.

Until now, most efforts to assess brain health have employed measures designed primarily for diagnosis or deficit detection, relying on normative comparisons and threshold-based criteria. While such measures are essential for identifying impairments, they fail to recognize that brain health is more than just the absence of brain compromise. Additionally, existing assessments tend to be screening-level scales or measure isolated pillars of brain health such as cognition^20,21,67,68^, socialization^69,70^, mental health^71^, physical fitness^72,73^, or sleep^74^. These approaches fail to capture how these components work in tandem to support overall brain health. In sum, this typical fragmented approach overlooks the dynamic and interrelated nature of brain health. In contrast, our findings suggest that incremental changes (gains or losses) in the personal holistic brain-behavioral profile can be regularly monitored, managed, and actioned using a platform that can be accessed remotely through online technology, including mobile apps.

Results from this study demonstrated the utility of a holistic, wellness-driven brain health measurement that is repeatable over time and actionable. The present findings advance our understanding of brain health *promotion* in two significant ways: (1) demonstrating the potential of a strategy-based executive function approach to facilitate behavior change and promote generalized, sustained benefits across multiple life domains and throughout adulthood and (2) underscoring the importance of leveraging accessible, tele-delivered tools to motivate self-agency of action and facilitate tailored application.

Beyond its impact on brain health, this study highlights the broader implications for overall health promotion. For instance, prior work with a large longitudinal sample (*n* = 10,855) of working-age adults in Finland demonstrated that not only can improved health behaviors promote gains in subjective well-being, but that this can be a bidirectional relationship, such that enhanced well-being can also further reinforce long-term health behaviors^75^. Optimizing brain health may serve as a foundation for holistic, lifelong health improvements.

### Limitations and future directions

This study has a number of limitations that should be considered when interpreting the findings. First, we acknowledge this is a single-arm interventional study and not a randomized control trial, so causal inferences may be limited. Prior randomized clinical trials (with combined totals of > 400 participants) using the SMART intervention have yielded findings demonstrating its effectiveness in promoting the types of gains observed, with some including evidence of corresponding neural changes^28–33,36–44^. The BrainHealth Project’s longitudinal approach allows a way to address the duration of the gains or detect early declines at an individual level. Future comparative studies with other brain health intervention types – including randomized controlled trials – could also be valuable.

Additionally, while a large study cohort has been enrolled to date, demographic diversity has been somewhat limited, most notably in racial, ethnic, and education level representation, which may limit the generalizability of the current findings to broader populations. Further, demographic and psychosocial factors may influence both engagement and outcomes, and our recruitment strategy likely enriched the sample for individuals already motivated to engage in proactive health activities. Efforts are underway to expand and enhance recruitment strategies to better reflect overall population demographics (age, sex, gender, race/ethnicity, level of education).

Another limitation is that the current collected data lack comprehensive information regarding participants’ medical histories and concurrent treatments that may impact an individual’s brain health trajectory. For example, participants’ lived experiences during the COVID-19 pandemic – including infection status, stress, and social isolation – could have influenced early engagement and outcomes. Although COVID-19 infection status was not directly queried during the early years, for subsequent waves of data collection, measures have been updated to include self-report of major health events, including COVID-19, along with expanded medical history. Also now incorporated are objective metrics of physical activity and sleep (e.g., wearable devices). The project also continues to expand ongoing investigations linking behavioral outcomes to neural changes through neuroimaging and other biomarkers. In particular, our network of laboratories (the BrainHealth Research Network) is refining and validating the BrainHealth Index against multiple neural markers derived from structural and functional magnetic resonance imaging (fMRI) analyses of associated changes on the BHI^26,76,77^. To address potential reporting bias in the psychometrically validated, self-reported components of the BHI, we corroborate these measures with objective indicators, including neural and hemodynamic changes^26^ and other biological metrics, such as sleep quality.

As with many online longitudinal studies, study attrition is a limitation – for this study sample, attrition rates across individual testing timepoints were relatively low, at 20% or lower. Moreover, because the study design accommodates self-paced engagement over a multi-year period, a portion of these instances likely represent transient pauses in participation rather than permanent study dropout. Future analyses will model these cyclical utilization trajectories to better differentiate temporary disengagement from true attrition, informing targeted retention strategies. Because of the need for at least two testing timepoints to evaluate change, this study’s sample did not include anyone from the BrainHealth Project with only a baseline assessment, so potential attrition within the first six-month period was not taken into account – which we estimate would bring the average attrition rate to around 43% for the broader longitudinal study cohort over the three years. This represents a comparable rate to what has been shown in previous longitudinal, population-based studies involving internet-based/eHealth platforms^78^. While attrition and lack of adherence have been longstanding barriers to successful health-behavior interventions^79^, similar attrition levels appear in long-term medication adherence^47^. Furthermore, even if only half of all users continue usage for three years or longer, the public health benefits of a platform accessible to millions of people will be enormous. Future work could examine whether shifts in individual brain health trajectories and/or utilization patterns precede study discontinuation, helping to inform targeted approaches to reduce participant dropout. That being said, we are actively engaged in adapting the platform to enhance engagement and long-term retention – for example, via more personalized follow-ups and community-building strategies.

## Conclusion

Given the brain’s central role in shaping who we are and how we function, these results emphasize the potential gains to the human brain health span achieved through promoting brain health from young adulthood to older ages with personalized and participatory practices. Science is clear that the time is urgent to expand medical practice and public health approaches to move away from a predominant brain disease focus toward a public health imperative that delivers brain health interventions. Such an approach will enable more people to realize their full potential over their life course, regardless of the presence or absence of brain issues. Health care providers can be on the forefront of brain health promotion by (1) adopting proven ways to *measure* growth in brain skills whatever one’s starting point and by (2) guiding their patients to protocols which will most *enhance* their brain skills with simple strategies. This effort parallels the advancements made in heart health over the past six decades, with the goal of optimizing each individual’s peak brain health span to match increasing longevity.

Finally, we propose that the potential societal and economic benefits of advancing brain health are significant. Proactively building stronger brain health and performance not only promotes individual well-being but also offers the prospect of reduced healthcare costs and increased productivity. As such, future research should explore the broader impact of scalable, precision brain health approaches on societal outcomes. This achievement will be possible if we make brain health a public health imperative – focusing on neuroplasticity, self-agency, and proactive growth while shifting from a reactive, deficit-based model.

## Electronic Supplementary Material

Below is the link to the electronic supplementary material.

Supplementary Material 1

## Data Availability

The datasets used and analyzed during the current study are available from the corresponding author upon reasonable request and with appropriate IRB approvals. The BrainHealth Platform is designed to enhance scientific reproducibility by administering all assessments through a standardized, version-controlled environment with consistent scoring logic and data processing pipelines. Immutable raw assessment records, automated scoring outputs, and detailed metadata are maintained to support auditability and verification of analytic methods. Approved research partners may be granted controlled access to both the curated datasets and the assessment environment to reproduce scoring and preprocessing workflows, conduct secondary analyses, replicate study protocols, or evaluate generalizability in nested or follow-on studies. All access is governed by institutional security, privacy, and compliance requirements.
