# Supplementary material for: Measuring and increasing the brain health span across adulthood: a public health imperative
Source: Sci Rep. 2026 May 2;16:20415. doi: 10.1038/s41598-026-51403-3 (PMC13328555; doi:10.1038/s41598-026-51403-3)
Supplement: Supplementary file 1 — Supplementary Material 1 [file 41598_2026_51403_MOESM1_ESM.docx]

**Supplementary Table S1.** Participant characteristics by baseline performance (quartile) groups

| Demographic  variables | Q1 | | | | Q2 | | | | Q3 | | | | Q4 | | | |
| --- | --- | --- | --- | --- | --- | --- | --- | --- | --- | --- | --- | --- | --- | --- | --- | --- |
| Age Group | 18-25 | 26-45 | 46-65 | > 65 | 18-25 | 26-45 | 46-65 | > 65 | 18-25 | 26-45 | 46-65 | > 65 | 18-25 | 26-45 | 46-65 | > 65 |
| Sample size (N) | 38 | 143 | 417 | 392 | 26 | 122 | 393 | 451 | 15 | 75 | 403 | 498 | 21 | 73 | 426 | 473 |
|  | n (%) | n (%) | n (%) | n (%) | n (%) | n (%) | n (%) | n (%) | n (%) | n (%) | n (%) | n (%) | n (%) | n (%) | n (%) | n (%) |
| Gender |  |  |  |  |  |  |  |  |  |  |  |  |  |  |  |  |
| Female | 24  (63.16) | 97  (67.83) | 335  (80.34) | 284  (72.45) | 18  (69.23) | 76  (62.30) | 315  (80.15) | 334  (74.06) | 11  (73.33) | 53  (70.67) | 329  (81.64) | 366  (73.49) | 13  (61.90) | 52  (71.23) | 350  (82.16) | 367  (77.59) |
| Male | 14  (36.84) | 46  (32.17) | 82  (19.66) | 108  (27.55) | 8  (3.77) | 46  (37.70) | 78  (19.85) | 117  (25.94) | 4  (26.67) | 22  (29.33) | 74  (18.36) | 132  (26.51) | 8  (38.10) | 21  (28.77) | 76  (17.84) | 106  (22.41) |
| Race |  |  |  |  |  |  |  |  |  |  |  |  |  |  |  |  |
| White | 22  (57.90) | 106 (74.13) | 358  (85.85) | 361  (92.09) | 20  (76.92) | 102  (83.61) | 361  (91.85) | 430  (95.34) | 12  (80.00) | 63  (84.00) | 363  (90.07) | 469  (94.18) | 18  (85.71) | 52 (71.23) | 391 (91.78) | 442 (93.45) |
| Asian | 7  (18.42) | 12  (8.39) | 23  (5.52) | 9  (2.30) | 2  (7.69) | 6  (4.92) | 14  (3.56) | 3  (0.67) | 2  (13.33) | 6  (8.00) | 14  (3.47) | 6  (1.20) | 2  (9.53) | 8 (10.96) | 14 (3.29) | 7 (1.48) |
| Black or African American | 3  (7.89) | 9  (6.29) | 6  (1.43) | 3  (0.77) | 0(0) | 3  (2.46) | 6  (1.53) | 5  (1.11) | 0(0) | 0(0) | 12  (2.98) | 8  (1.61) | 1 (4.76) | 3 (4.11) | 10 (2.35) | 7 (1.48) |
| Native American / Alaska Native | 0 (0) | 0 (0) | 0 (0) | 1 (0.25) | 0(0) | 0(0) | 0(0) | 0(0) | 0(0) | 0(0) | 0(0) | 1 (0.20) | 0(0) | 1 (1.37) | 0(0) | 0(0) |
| Native Hawaiian / Pacific Islander | 0 (0) | 1 (0.70) | 0 (0) | 1 (0.25) | 0(0) | 0(0) | 0(0) | 0(0) | 0(0) | 1 (1.33) | 0(0) | 0(0) | 0(0) | 0(0) | 0(0) | 0(0) |
| Other | 5  (13.16) | 6  (4.20) | 9  (2.16) | 10  (2.55) | 1  (3.85) | 7  (5.74) | 6  (1.53) | 4  (0.89) | 0(0) | 2 (2.67) | 5  (1.24) | 6  (1.20) | 0(0) | 3 (4.11) | 5 (1.17) | 7 (1.48) |
| Multiple races | 1  (2.63) | 9  (6.29) | 21  (5.04) | 7  (1.79) | 3  (11.54) | 4  (3.28) | 6  (1.53) | 9  (1.99) | 1  (6.67) | 3  (4.00) | 9  (2.24) | 8  (1.61) | 0(0) | 6 (8.22) | 6 (1.41) | 10 (2.11) |
| Hispanic or Latino |  |  |  |  |  |  |  |  |  |  |  |  |  |  |  |  |
| Yes | 7 (18.42) | 17 (11.89) | 22 (5.28) | 5  (1.28) | 4 (15.38) | 15 (12.30) | 34 (8.65) | 7 (1.55) | 0(0) | 8 (10.67) | 21 (5.21) | 12 (2.41) | 1 (4.76) | 11 (15.07) | 20 (4.69) | 13 (2.75) |
| No | 31 (81.58) | 126 (88.11) | 395 (94.72) | 387 (98.72) | 22 (84.62) | 107 (87.70) | 359 (91.35) | 444 (98.45) | 15 (100) | 67 (89.33) | 382 (94.79) | 486 (97.59) | 20 (95.24) | 62 (84.93) | 406 (95.31) | 460 (97.25) |
| Education |  |  |  |  |  |  |  |  |  |  |  |  |  |  |  |  |
| Pre Bachelor | 17 (44.74) | 23 (16.08) | 87 (20.86) | 86 (21.94) | 7 (26.92) | 17 (13.93) | 50 (12.72) | 61 (13.52) | 4 (26.67) | 9 (12.00) | 31 (7.69) | 58 (11.65) | 4 (19.05) | 3 (4.11) | 34 (7.98) | 41 (8.67) |
| Bachelor | 17 (44.74) | 56 (39.16) | 168 (40.29) | 129 (32.91) | 12 (46.16) | 51 (41.80) | 157 (39.95) | 142 (31.49) | 7 (46.66) | 31 (41.33) | 173 (42.93) | 159 (31.93) | 8 (38.09) | 25 (34.25) | 168 (39.44) | 133 (28.12) |
| Post Bachelor | 4 (10.52) | 64 (44.76) | 162 (38.85) | 177 (45.15) | 7 (26.92) | 54 (44.27) | 186 (47.33) | 248 (54.99) | 4 (26.67) | 35 (46.67) | 199 (49.38) | 281 (56.42) | 9 (42.86) | 45 (61.64) | 224 (52.58) | 299 (63.21) |
| Annual household income |  |  |  |  |  |  |  |  |  |  |  |  |  |  |  |  |
| Less than $20,000 | 12 (31.59) | 8 (5.60) | 25 (6.00) | 26 (6.63) | 5 (19.23) | 4 (3.28) | 10 (2.54) | 13 (2.88) | 3 (20.00) | 3 (4.00) | 3 (0.74) | 10 (2.00) | 2 (9.52) | 1 (1.37) | 5 (1.15) | 4 (0.84) |
| $20,000 - $39,999 | 6 (15.79) | 12 (8.39) | 37 (8.87) | 60 (15.30) | 2 (7.69) | 5 (4.10) | 23 (5.85) | 48 (10.64) | 0 (0) | 2 (2.67) | 15 (3.72)) | 27 (5.42) | 1 (4.76) | 2 (2.74) | 9 (2.11) | 34 (7.19) |
| $40,000 - $59,999 | 3 (7.89) | 12 (8.39) | 47 (11.27) | 59 (15.05) | 0 (0) | 14 (11.48) | 33 (8.40) | 64 (14.20) | 3 (20.00) | 5 (6.67) | 24 (5.95) | 60 (12.05) | 2 (9.52) | 4 (5.48) | 22 (5.16) | 57 (12.05) |
| $60,000 - $99,999 | 4 (10.53) | 36 (25.17) | 87 (20.86) | 102 (26.02) | 6 (23.08) | 22 (18.03) | 68 (17.30) | 124 (27.50) | 2 (13.33) | 13 (17.33) | 90 (22.33) | 155 (31.12) | 5 (23.81) | 13 (17.81) | 63 (14.79) | 108 (22.83) |
| $100,000 - $250,000 | 9 (23.68) | 54 (37.76) | 160 (38.37) | 101 (25.77) | 10 (38.46) | 57 (46.72) | 177 (45.04) | 130 (28.82) | 4 (26.67) | 39 (52.00) | 174 (43.18) | 169 (33.94) | 8 (38.10) | 35 (47.94) | 190 (44.60) | 180 (38.06) |
| More than $250k | 3 (7.89) | 19 (13.29) | 39 (9.35) | 13 (3.32) | 1 (3.85) | 18 (14.75) | 67 (17.05) | 29 (6.43) | 1 (6.67) | 11 (14.66) | 78 (19.36) | 33 (6.63) | 3 (14.29) | 15 (20.55) | 113 (26.53) | 53 (11.21) |
| Not Available/Prefer not to answer | 1 (2.63) | 2 (1.40) | 22 (5.28) | 31 (7.91) | 2 (7.69) | 2 (1.64) | 15 (3.82) | 43 (9.53) | 2 (13.33) | 2 (2.67) | 19 (4.72) | 44 (8.84) | 0 (0) | 3 (4.11) | 24 (5.63) | 37 (7.82) |
| Self-reported psychiatric diagnoses |  |  |  |  |  |  |  |  |  |  |  |  |  |  |  |  |
| None | 22 (57.90) | 85 (59.44) | 278 (66.67) | 260 (66.33) | 20 (76.92) | 99 (81.15) | 331 (84.22) | 360 (79.82) | 14 (93.33) | 60 (80.00) | 346 (85.86) | 443 (88.96) | 18 (85.71) | 69 (94.52) | 400 (93.90) | 443 (93.66) |
| One | 11 (28.95) | 36 (25.18) | 85 (20.38) | 85 (21.68) | 6 (23.08) | 17 (13.93) | 42 (10.69) | 72 (15.97) | 1 (6.67) | 12 (16.00) | 45 (11.16) | 46 (9.24) | 3 (14.29) | 4 (5.48) | 22 (5.16) | 26 (5.49) |
| Multiple | 5 (13.16) | 22 (15.39) | 54 (12.95) | 47 (11.99) | 0 (0) | 6 (4.92) | 20 (5.09) | 19 (4.21) | 0 (0) | 3 (4.00) | 12 (2.98) | 9 (1.80) | 0 (0) | 0 (0) | 4 (0.94) | 4 (0.85) |
| Self-reported medical conditions |  |  |  |  |  |  |  |  |  |  |  |  |  |  |  |  |
| None | 31 (81.58) | 122 (85.32) | 280 (67.15) | 236 (60.20) | 24 (92.31) | 113 (92.62) | 280 (71.25) | 327 (72.51) | 13 (86.67) | 69 (92.00) | 318 (78.91) | 375 (75.30) | 20 (95.24) | 70 (95.89) | 353 (82.86) | 385 (81.40) |
| One | 5 (13.16) | 15 (10.49) | 95 (22.78) | 100 (25.51) | 2 (7.69) | 6 (4.92) | 80 (20.35) | 91 (20.18) | 2 (13.33) | 5 (6.67) | 63 (15.63) | 98 (19.68) | 0 (0) | 2 (2.74) | 56 (13.15) | 69 (14.59) |
| Multiple | 2 (5.26) | 6 (4.20) | 42 (10.07) | 56 (14.29) | 0 (0) | 3 (2.46) | 33 (8.40) | 33 (7.31) | 0 (0) | 1 (1.33) | 22 (5.46) | 25 (5.02) | 1 (4.76) | 1 (1.37) | 17 (3.99) | 19 (4.01) |

**Supplementary Table S2.** Numbers of completed assessments and drop-offs during the 3-year study period across longitudinal timepoints by baseline performance (quartile) group

| Cycle number | Q1 | | Q2 | | Q3 | | Q4 | | Completed total |
| --- | --- | --- | --- | --- | --- | --- | --- | --- | --- |
|  | Completed | Drop-offs* | Completed | Drop-offs* | Completed | Drop-offs* | Completed | Drop-offs* |  |
| T1 | 990 | - | 992 | - | 991 | - | 993 | - | 3966 |
| T2 | 990 | - | 992 | - | 991 | - | 993 | - | 3966 |
| T3 | 570 | 209 | 640 | 206 | 643 | 190 | 648 | 201 | 2501 |
| T4 | 349 | 53 | 431 | 50 | 413 | 47 | 470 | 60 | 1663 |
| T5 | 208 | 9 | 304 | 14 | 297 | 16 | 344 | 14 | 1153 |
| T6 | 141 | 0 | 195 | 4 | 188 | 4 | 240 | 7 | 764 |
| T7 | 89 | 0 | 115 | 1 | 116 | 0 | 158 | 1 | 478 |

**Note: Drop-offs* are distinguished from those who are not yet due for their assessment at that timepoint and are defined as having an assessment remaining *due but incomplete* for longer than a year, based on the date assigned. Because of the study's continuous, multi-year design, drop-offs may reflect episodic pauses in engagement rather than permanent attrition; data current as of sample date (March 1, 2025)

**Supplementary Table S3.** Longitudinal gradient metrics by baseline performance (quartile) groups

|  | Overall BHI | | Connectedness | | Emotional Balance | | Clarity | |
| --- | --- | --- | --- | --- | --- | --- | --- | --- |
|  | gradient | SE | gradient | SE | gradient | SE | gradient | SE |
| Q1 | 17.94 | 0.92 | 20.86 | 0.76 | 14.27 | 0.58 | 16.93 | 1.42 |
| Q2 | 10.97 | 0.92 | 13.64 | 0.76 | 7.54 | 0.58 | 12.41 | 1.42 |
| Q3 | 6.21 | 0.92 | 9.73 | 0.76 | 5.30 | 0.58 | 10.75 | 1.42 |
| Q4 | 6.51 | 0.92 | 9.85 | 0.76 | 0.54 | 0.58 | 7.31 | 1.42 |


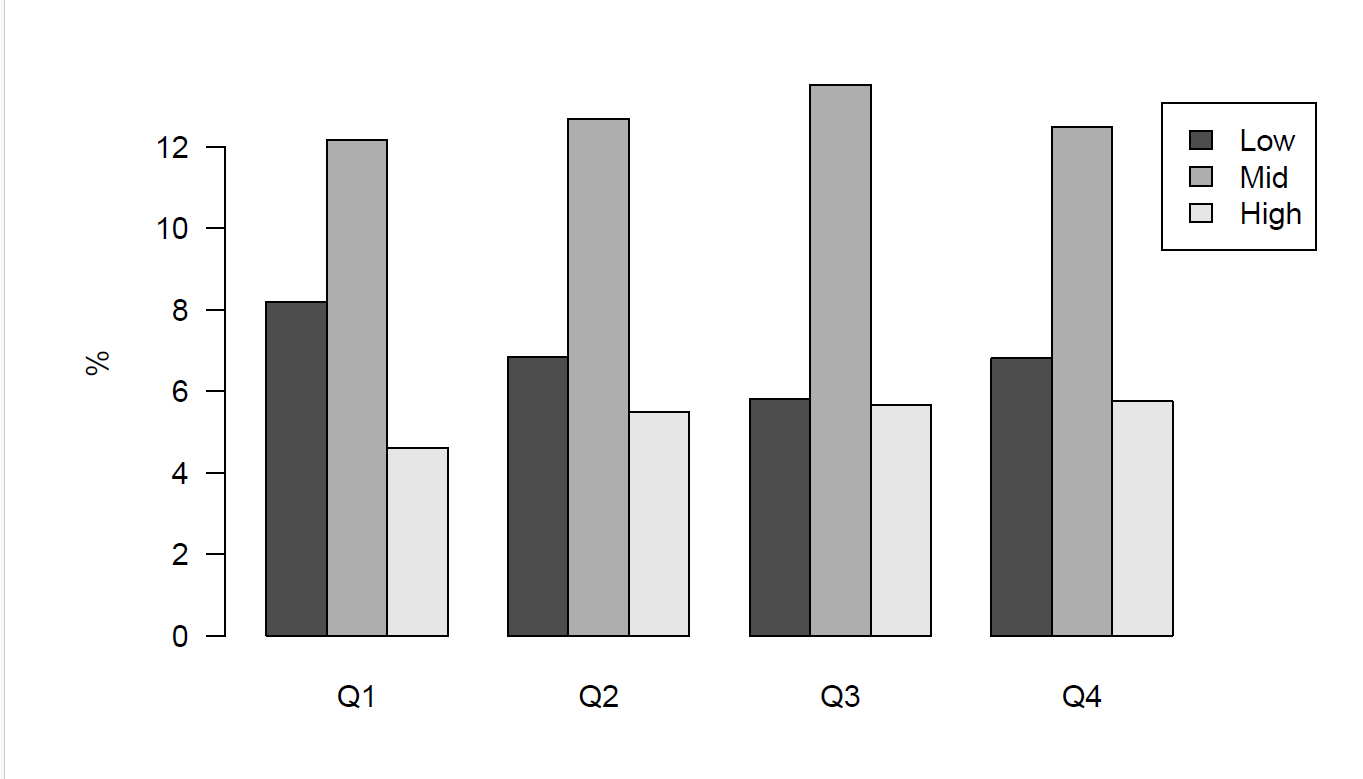


**Supplementary Figure S4.** Percentage of participants within each utilization group broken out by baseline performance (quartile) group
